# MTC-NET: A Multi-Channel Independent Anomaly Detection Method for Network Traffic

**DOI:** 10.3390/biomimetics9100615

**Published:** 2024-10-10

**Authors:** Xiaoyong Zhao, Chengjin Huang, Lei Wang

**Affiliations:** 1 School of Information Management, Beijing Information Science and Technology University, Beijing 100192, China; zhaoxiaoyong@bistu.edu.cn (X.Z.); wanglei575882@163.com (L.W.); 2 Information Systems Research Institute, Beijing Information Science and Technology University, Beijing 102206, China

**Keywords:** network traffic anomaly detection, multi-channel Transformer–CNN, intrusion detection, network flow, deep learning, artificial intelligence

## Abstract

In recent years, deep learning-based approaches, particularly those leveraging the Transformer architecture, have garnered widespread attention for network traffic anomaly detection. However, when dealing with noisy data sets, directly inputting network traffic sequences into Transformer networks often significantly degrades detection performance due to interference and noise across dimensions. In this paper, we propose a novel multi-channel network traffic anomaly detection model, MTC-Net, which reduces computational complexity and enhances the model’s ability to capture long-distance dependencies. This is achieved by decomposing network traffic sequences into multiple unidimensional time sequences and introducing a patch-based strategy that enables each sub-sequence to retain local semantic information. A backbone network combining Transformer and CNN is employed to capture complex patterns, with information from all channels being fused at the final classification header in order to achieve modelling and detection of complex network traffic patterns. The experimental results demonstrate that MTC-Net outperforms existing state-of-the-art methods in several evaluation metrics, including accuracy, precision, recall, and F1 score, on four publicly available data sets: KDD Cup 99, NSL-KDD, UNSW-NB15, and CIC-IDS2017.

## 1. Introduction

The accelerated evolution of the Internet has resulted in the emergence of more sophisticated network security challenges, which have proven to be challenging for traditional detection methods to effectively address. The identification of anomalous network traffic has emerged as a pivotal strategy for safeguarding network resources from potential attacks.

In previous research, scholars have employed a range of techniques to identify anomalous traffic, including rule-based and statistical learning-based methods. Rule-based methods are constrained by their reliance on deterministic rules and expert knowledge, which limits their scope and applicability [1,2]. Statistical learning-based methods are dependent on the extraction of features through manual processes. In recent years, there has been a notable emergence of deep learning-based detection methods, with the Transformer model being a prominent example. Its efficacy in processing sequential data has led to its widespread use in network traffic anomaly detection [3,4].

Although the Transformer model demonstrates efficacy in capturing long-range dependencies in sequential data, its direct application to network traffic data in the presence of noise frequently results in a decline in detection performance due to the interference of noise between sequential, non-homogeneous data. Furthermore, existing methodologies tend to disregard the capacity to capture local features when confronted with intricate network traffic patterns, and are incapable of fully leveraging the subtle patterns and temporal features inherent to network traffic data. It is therefore necessary to develop a novel and effective method that can effectively capture global dependencies and enhance the sensitivity to local features, with the aim of improving the accuracy and robustness of network traffic anomaly detection.

In recent years, bionics has played an important role in advancing artificial intelligence technology. Bionics has provided important inspiration and guidance for the development of modern technologies by studying and imitating various mechanisms and models of living things themselves in nature. Applied to deep learning and network traffic anomaly detection, bionics has inspired the concept of multi-channel information processing, similar to the way a biological brain processes signals from multiple different sensory channels, such as vision, hearing, etc., simultaneously in complex environments.

In this study, the proposed MTC-Net model adopts a multi-channel strategy, inspired by bionics, to mimic the separate processing of information from different sensory channels in biological systems by splitting the channels to process information from different dimensions of network traffic, which helps the model to better recognise and detect anomalous patterns. In addition, similar to the adaptive behavior in biological systems (e.g., organisms adjust their response strategies in different environments), the multi-channel structure of MTC-Net can adaptively learn to adjust the information weight of each channel during training according to the different characteristics of the network traffic, which can help the model flexibly adjust its detection strategy when facing different types of cyber attacks to improve the accuracy and robustness of detection.

In MTC-Net, the network traffic sequence is regarded as a multidimensional time series and is decomposed into multiple unidimensional time series, thereby effectively isolating the mutual interference between different dimensions. The channel independence of this approach ensures that the features in each dimension are not contaminated by noise or information in other dimensions during the modelling process, thereby enhancing the accuracy of detection. Furthermore, the model combines local and global feature extraction, analogous to multi-scale processing in natural systems, which enables more effective identification of anomalous traffic patterns in complex and noisy environments.

To further enhance the performance of the model, we adopt the patch strategy, which involves the local segmentation of each 1D time series into multiple patches. This approach has the dual benefit of reducing the computational complexity of the model and allowing it to retain key local semantic information when processing long time series. In the backbone network design, the advantages of Transformer and CNN are combined to enhance the model’s ability to capture complex patterns. The design of MTC-Net not only reduces computational complexity while maintaining the capture of global dependencies, but also significantly enhances the model’s sensitivity to local features.

## 2. Backgroud and Related Work

In previous studies, researchers have proposed numerous anomaly detection algorithms for network traffic. Intrusion detection models can be classified as either binary or multi-classification, depending on the number of classification categories [5,6,7,8,9]. Binary classification methods typically construct a model of normal traffic and categorise all traffic that deviates from the normal model as attacks. In contrast, multi-classification models identify the specific distribution of each type of attack. The initial approaches were constrained in their capabilities and prone to inaccuracy. As computing power grew, models based on machine learning and deep learning gradually gained prominence.

### 2.1. Previous Intrusion Detection Methods

Previous intrusion detection methodologies have placed significant reliance on statistical analysis and conventional machine learning techniques. For example, Tsai and Lin [10] proposed a hybrid learning model based on triangular region nearest neighbours, which enables the detection of cyber attacks by calculating the distances between sample points. Bostani and Sheikhan [11] improved the optimal path forest (OPF) algorithm and combined it with the concepts of unsupervised learning and social networks to enhance the performance of intrusion detection systems (IDSs). Furthermore, Al-Yaseen et al. [12] proposed a packing feature selection method that combines differential evolutionary algorithms and extreme learning machines, thereby enhancing the accuracy and efficiency of the system. However, these methods typically address individual data streams, yet they do not fully account for the sequential nature inherent to network traffic.

The advent of ChatGPT [13] has heralded a new era of artificial intelligence, characterised by the emergence of large models based on the Transformer architecture [14]. This sequence-to-sequence (seq2seq) architecture enables the capture of remote dependencies between disparate elements within a sequence, obviating the necessity for additional feature engineering. Initially, Transformer was employed primarily for natural language processing tasks. However, recent research has demonstrated its efficacy in capturing intricate patterns and relationships in data across diverse sequence types, including images and speech. The Transformer has demonstrated a robust capacity for adaptation, establishing itself as a dominant paradigm in the field of deep learning.

In recent years, there has been a proliferation of methods based on the Transformer model that have been proposed and applied to the field of network traffic anomaly detection. Wu et al. [3] proposed a method called RTIDS, which used the structure of a variant stacked Transformer encoder–decoder to build an intrusion detection system. This approach achieved efficient processing and accurate classification of network traffic data. Yun et al. [15] combined the advantages of the graph neural network and the Transformer model, thereby capturing the relationship and topology between data in complex network environments more effectively. This approach provided new ideas for intrusion detection systems. Yang et al. [16] also proposed an intrusion detection system based on the improved Vision Transformer model approach, where they optimised the structure and parameter settings of the Transformer, thus achieving a significant improvement in performance on the intrusion detection task. This suggests that the Transformer model has a wide range of potential applications in the field of intrusion detection and is well suited to dealing with complex network attack behaviours. The Manocchio et al. [4] flow Transformer framework is an effective method for detecting and classifying anomalous behaviours in a flow data set, due to its modular design.

### 2.2. Our Work

Previous research on network traffic has frequently neglected its sequential nature [17], instead concentrating solely on the attributes of individual data streams for the purpose of detection and classification. Given the sequential nature of network traffic, a Transformer-based model that effectively captures long-term dependencies through a self-attention mechanism is proposed as a means of improving anomaly detection. This approach is particularly suited to coping with complex network attacks that require consideration of time series behaviours, such as the SlowLoris attack [18].

This paper proposes a multi-channel network traffic anomaly detection model, designated Multi-Channel Transformer–CNN (MTC-Net). A Patch strategy was employed to achieve channel independence between dimensions, thereby enabling the model to concentrate on capturing anomaly patterns and timing features present in each dimension. Concurrently, a convolutional neural network and maximum pooling strategy are employed to downscale the dimensionally expanded channels. Ultimately, the classification header facilitates the integration and interaction of features across multiple channels.

In conclusion, the model demonstrates superior performance across all evaluation metrics. A comprehensive evaluation was conducted on four publicly available network intrusion training sets: KDD99 [19], NSL-KDD [20], UNSW-NB15 [21], and CICIDS2017 [22]. Additionally, relevant ablation experiments were performed to validate the effectiveness of each module. The aforementioned experiments substantiate the superiority and reliability of the proposed MTC model for the detection of anomalies in network traffic.

## 3. Proposed Methodology

**Figure 1:**
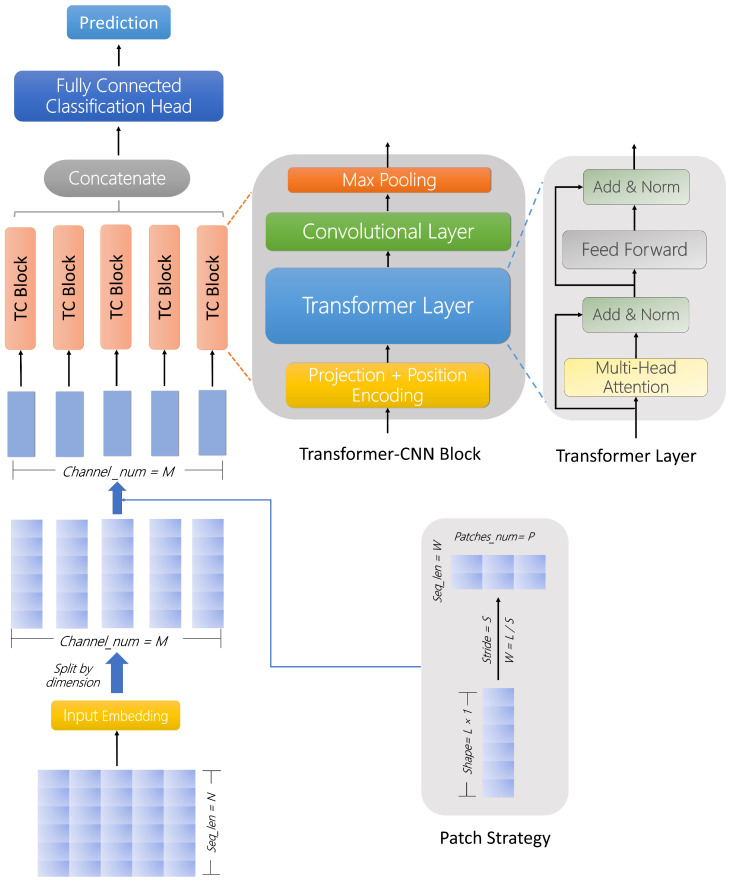
The MTC model comprises an input embedding layer, M parallel Transformer–CNN (TC) modules (M equals the number of channels), a fully connected layer classification header, and an output layer. The TC modules comprise a projection layer and position coding, a Transformer encoder, a convolutional neural network (CNN) layer, and a maximum pooling layer. The figure also illustrates a “Patch Strategy”, which divides a one-dimensional sequence of length, L, into patches of shape P × W based on a stride, S. The patches can overlap, while the example in the figure shows a case where the patches do not overlap. When the patches do not overlap, S = P.

Previous research has demonstrated that when Transformer is directly applied to multidimensional time series data, there are issues of mutual interference and noise between data of different dimensions. In light of the aforementioned issues, this paper proposes a multi-channel network traffic anomaly detection model, MTC-Net, which combines Transformer with a convolutional neural network (CNN) [23]. The model decomposes the network traffic sequence into multiple one-dimensional time series and introduces the Patch strategy, which enables the model to achieve the best performance in terms of both local feature sensitivity and global feature capturing ability. The backbone network incorporates essential elements, including the Transformer encoder and CNN, as illustrated in Figure 1.

### 3.1. Design of Model Architecture

The network traffic sequence is a multi-channel signal, and each Transformer input token can be represented by data from a single channel or multiple channels. In this context, channel independence signifies that each input token contains information from a single channel only. To enhance the model’s capacity to discern local features, we propose the incorporation of the Patch strategy. This strategy employs the concept of image segmentation, whereby images are divided into smaller units (patches), as a means of processing sequence data. In MTC-Net, the term “Patch” denotes the partitioning of each sub-sequence into a series of discrete segments of a fixed length. These segments are then employed as input tokens for the Transformer, enabling the effective capture of short-term dependencies and local patterns within the sequence [24].

In MTC-Net, the network traffic data are divided into multiple sub-sequences, each of which is encoded with Transformer. This process is an effective means of capturing potential dependencies in sequences, both short-term and long-term. Furthermore, the incorporation of positional encoding guarantees that the model is able to accurately comprehend the sequence ordering of these sub-sequences during processing. The aforementioned encoding enables the model to demonstrate enhanced resilience and precision in the context of network attacks characterised by long-range dependencies, such as DDoS attacks.

#### 3.1.1. Patch Strategy and Channel Independence

A network traffic sequence is a multi-channel signal, and each Transformer input token can be represented by data from a single channel or multiple channels. Channel mixing refers to the process of combining vectors representing all sequence features into a single embedding space, thereby mixing the information. This is in contrast to channel independence, which implies that each input token only contains information from a single channel [25]. To enhance the model’s capacity to discern local features, we propose the introduction of the Patch strategy. This strategy employs the concept of image segmentation, whereby images are divided into smaller units (patches), as a means of processing sequence data. In MTC-Net, the term “Patch” denotes the partitioning of each sub-sequence into a series of discrete segments of a fixed length. These segments are then employed as input tokens for the Transformer, thereby achieving channel independence. This approach facilitates the effective capture of short-term dependencies and local patterns within the sequence.

The introduction of this strategy offers multiple advantages. Firstly, the Patch strategy reduces the number of tokens entered into the Transformer each time, thus reducing the computational complexity. Secondly, the positional encoding ensures that the sequential information of the sequences is preserved. Thirdly, the application of the Patch strategy allows the model to better detect local anomalies in network traffic [26], such as in the attack patterns that occur with high frequency within a short period of time. This approach can be employed to identify network traffic anomalies characterised by local irregularities.

#### 3.1.2. Combining Convolutional Neural Networks (CNNs)

Following the implementation of the Transformer encoder, we introduce a convolutional neural network (CNN) for the purpose of reducing the dimensionality of the data set and facilitating the extraction of additional features. The CNN is capable of handling high-dimensional data sets and of capturing local patterns with great efficacy. The convolution operation enables the CNN to effectively identify local features in the data, while the pooling operation facilitates the reduction in feature dimensions. This avoids the accumulation of excessive data dimensions in the input classification header, thereby enhancing the robustness of the model.

In MTC-Net, each sub-sequence is processed by both the Transformer and CNN, resulting in the generation of a feature vector. Subsequently, the feature vectors of all sub-sequences are merged to create a comprehensive feature representation, which is then input to the fully connected layer. This allows for the integration of features extracted from all channels, facilitating the final classification operation. This combination enables the model to capture global dependencies and extract key local features, thereby significantly enhancing the accuracy and robustness of anomaly detection.

This design offers several significant advantages. The Patch strategy enables the model to reduce the length of the input sequence, thereby reducing the computational complexity and improving computational efficiency. This allows MTC-Net to maintain a low level of computational resource usage when processing large data sets.

The introduction of the Transformer encoder enables the model to effectively capture long temporal dependencies, which is a significant advancement in the field of machine learning. This is particularly crucial for anomaly detection in network traffic data, as certain anomalous behaviours may manifest themselves as minor alterations over an extended timespan. Consequently, accurate detection can only be achieved through the analysis of long time series.

The modular design of MTC-Net allows for flexibility in processing, with each Patch capable of independent operation and channel independence ensuring scalability. This adaptability to data sets of varying sizes and application scenarios is a key advantage of MTC-Net.

### 3.2. MTC-Net

At present, there are still a relatively limited number of studies that can effectively address the problem of network traffic sequence modelling, and thus there is a large gap in this field. Transformer has emerged as a powerful option in network traffic sequence modelling research due to its inherent ability to process sequence data and capture complex relationships between elements at different locations in the sequence.

In our study, we propose a deep neural network for network traffic anomaly detection, called MTC-Net, as shown in Figure 1. MTC-Net consists of three main modules: a data processing module, a Transformer–CNN (**TC**) module, and an output prediction module.

First, we pre-process the original network traffic data set to ensure the high quality of the input data. Taking the CIC-IDS2017 data set as an example, the pre-processing mainly consists of the following three steps:

Attribute numerisation: First, the string type attributes in the data set are converted to numeric types. This step ensures that the data can be correctly processed and understood by the model.

Data normalisation: To normalise the data set, a min-max normalisation method is used, which maps the value ranges of all variables to between 0 and 1. This helps to eliminate the negative effects of differences in the magnitude of different variables on model training, thus improving the convergence speed and performance of the model.

Timestamp extraction and serialisation: We extract the raw timestamp information from the data set for additional temporal embedding before the sequence is fed into the model, and use full-connectivity embedding directly for data sets without timestamp information. Timestamps are key time series data that can help the model to capture temporal dependencies and sequential information. After pre-processing the data, we perform a serialisation operation on the processed data set to generate the flow-serialised data.

Finally, we obtain the original sequence *L*: $\left(x_{1},\ldots ,x_{L}\right)$ with window size *L*, where each $x_{t}$ is a vector of dimension *M*. Then, we need to embed the sequence and then feed the sequence into the mesh. First, we need to split the sequence into *M* sub-sequences and input each sub-sequence as a channel into the **TC** module for individual feature extraction.

In the **TC** module, we first process the sub-sequence $x^{\left(i\right)}\in R^{1\times L}$, reshape the sequence according to the step size *S*, and generate the sequence $x^{\left(i\right)}\in R^{P\times W}$ of length *W*. In the process, each Patch can be chosen to overlap or not. Here, *P* denotes the number of patches generated. By using patches, the number of input tokens can be reduced from *L* to about L/S. This reduces the space and time complexity of the attention weighting with respect to $S^{2}$. Immediately after that, the sequences are mapped into a *D*-dimensional high-dimensional potential space, $x^{\left(i\right)}\in R^{P\times D}$, using a projection layer via a trainable linear projection, $W_{p}\in R^{W\times D}$. To capture temporal relationships and local features in the sequences, we introduce learnable additional positional encodings to ensure that the model is able to understand the order of the Patch, thus enhancing the model’s ability to understand the data.

The processed sub-sequences are fed into the Transformer layer separately. In the multi-head attention mechanism, each head, h=1,…,H, transforms the input into query matrix $Q=XW^{Q}$, key matrix $K=XW^{K}$ and value matrix $V=XW^{V}$, and then obtains the attention output:

(1) $$\mathrm{Attention}\left(Q,K,V\right)=\mathrm{softmax}\left(\frac{QK^{T}}{\sqrt{d_{k}}}\right)V$$

As shown in Figure 1, the output structure is fed into the feed-forward neural network (FFN) using residual connection and regularisation operations, and another residual connection is performed. To further extract the effective features and reduce the dimension of the expanded features, after processing by Transformer, we feed the output into the convolutional neural network (CNN) for convolution operation, and perform Max Pooling on the convolved result.

(2) $$Y\left(i,j\right)=\sum_{m=0}^{M-1}\sum_{n=0}^{N-1}X\left(i+m,j+n\right)\cdot W\left(m,n\right)+b$$

- Y(i,j) denotes the elements of the convolution output feature matrix;
- *X* denotes the input feature map;
- *W* denotes the convolution kernel;
- *b* denotes the bias term;
- *M* and *N* are the convolution kernel height and width, respectively.

(3) $$Z\left(i,j\right)=\max_{0\leq m<M,0\leq n<N}Y\left(s_{i}+m,s_{j}+n\right)$$

- Z(i,j) denotes the element of the pooled output feature matrix;
- *Y* denotes the input feature map after convolution;
- $s_{i}$ and $s_{j}$ are the starting positions of the pooling window;
- *M* and *N* are the height and width of the pooling window, respectively.

We combine the outputs of all the sub-sequences processed by the **TC** module to obtain a complete sequence, $z=\mathrm{concat}(z_{1},\ldots ,z_{M})$, for the next classification operation. By integrating the outputs of the sub-sequences processed by the **TC** module, the model fully exploits the information of each channel and transforms this information into outputs through the fully connected layer to obtain the final prediction results.

## 4. Experimental Methods

We conducted a series of experiments to validate the effectiveness of the proposed MTC-Net. In this section, we first describe the data sets used and the data pre-processing, and finally present our experimental results. We conducted anomaly detection experiments using four public network traffic data sets, KDD Cup 99, NSL-KDD, UNSW-NB15 and CIC-IDS2017, and analysed them in comparison with some previous anomaly detection methods. To validate the performance of the model, we compared the MTC-Net model with some state-of-the-art anomaly detection methods. In addition, to show the performance of our proposed model on different tasks and data, we also performed multi-classification experiments on these data sets.

### 4.1. Data Set

KDD Cup 99 [19] was first proposed in the 1999 KDD Cup competition. The KDD Cup 99 data set uses the raw data from the DARPA 1998 data set, which was pre-processed from the DARPA 98 data set to extract a single record in terms of a “connection”. The data set contains up to 41 features covering all aspects of network connectivity, including connection duration, service type, transport layer protocols, and so on. The data set mainly contains 5 different types of attacks, covering common network intrusion behaviours such as denial of service attacks, brute force attacks, etc.

NSL-KDD [20] is a revised version based on the KDD Cup 99 data set. The NSL-KDD data set is improved to address the problems of highly unbalanced distribution of attack types and the presence of a large amount of redundant data in the KDD Cup 99. The NSL-KDD data set removes a large amount of duplicate data and balances the distribution of different data, thus improving the quality of the data set.

UNSW-NB15 [21] is a traditional network intrusion detection data set published in 2015. The data set has nine types of attacks, namely fuzzers, analysis, backdoors, DoS, exploits, generic, reconnaissance, shellcode and worms. The UNSW-NB15 data set contains 42 attributes. In addition, UNSW-NB15 contains two data subsets, one for model training and the other for model testing.

The CIC-IDS2017 [22] data set contains some of the latest common attacks that generate real-world-like data (PCAP). It also includes the results of network traffic analysis using CICFlowMeter, which contains tagged flows based on timestamps, source and destination IPs, source and destination ports, and protocols and attacks (CSV files). The data set includes a variety of attack types such as brute force, DDoS, web attacks, infiltration and port scan.

### 4.2. Data Pre-Processing

Before training, we performed data cleaning on the original data set, dealing with the missing values and outliers present in the data to ensure the completeness and accuracy of the data. Since anomaly detection is a classification task, we need to convert text labels into numerical values that the model can understand. Therefore, we need to encode the labels of the attack types in the data set and map each attack type to a corresponding numeric code. For this purpose, we created a mapping table to map attack types to numbers in order to encode text labels to numbers, e.g., converting the category “Normal” to “0”.

In order to pre-process the attributes of the data set, we carried out the following two main tasks:

1. **Digitising the attributes:** Some non-numeric features in the data set, such as “http” and “smtp”, which cannot be fed directly into the neural network, need to be converted into numeric form so that the data can be fed into the neural network. Therefore, we used integers starting from 1 to digitally encode the features of non-numerical types.
2. ***Min-Max* Normalisation:** Input feature values of different dimensions contribute differently to the final contribution of the model, and if the raw data are fed directly into the model, it may lead to bias during model training in favour of dimensions with larger feature values. Therefore, we used the min-max normalisation method to scale the data range to [0, 1], which helped to maintain some numerical comparability and improved the stability and speed of backpropagation. The ***min-max*** normalisation formula is defined as follows:
  (4) $$x^{\prime }=\frac{x-\min(x)}{\max(x)-\min(x)}$$
3. **Up-sampling:** In the multi-classification experiments, we up-sampled a very small number of samples that were too sparse, such as the “Heartbleed” class in the CIC-IDS2017 data set, which was up-sampled after dividing the training set, so that its sample number was increased tenfold and distributed across the training set.

### 4.3. Evaluation Metrics

This subsection defines the performance metrics used to evaluate the different techniques compared in this paper, as well as the formulas that specify their calculation. First, we need to understand the following concepts:

- **TP:** positive samples predicted by the model as positive classes;
- **TN:** negative samples predicted by the model to be in the negative category;
- **FP:** negative samples predicted by the model to be in the positive category;
- **FN:** Positive samples predicted by the model to be in the negative category.

The following are the evaluation metrics we used:

**Accuracy:** The ratio of the total number of samples correctly classified by the model to the number of all samples, i.e.,

(5) $$\mathrm{Accuracy}=\frac{TP+TN}{TP+FP+TN+FN}$$

**Precision:** The proportion of all samples correctly classified by the model to the number of samples whose true values are also correctly classified, i.e.,

(6) $$\mathrm{Precision}=\frac{TP}{TP+FP}$$

**Recall:** The proportion of samples with positive true values predicted by the model to the total number of samples with positive true values, i.e.,

(7) $$\mathrm{Recall}=\frac{TP}{TP+FN}$$

**F1 score:** takes into account both the precision and recall of the samples and is the harmonic mean of precision and recall, i.e.,

(8) $$F1=2\times \frac{\mathrm{Precision}\times \mathrm{Recall}}{\mathrm{Precision}+\mathrm{Recall}}$$

### 4.4. Loss Function

The loss function we chose was the cross-entropy loss, which measures the difference between two probability distributions and is often used to assess the agreement between the probability distribution predicted by the model and the probability distribution of the true labels. It is therefore often used in classification tasks. The cross-entropy loss function is defined as follows:

(9) $$L=-\frac{1}{n}\sum_{i=1}^{n}\sum_{c=1}^{C}y_{i,c}\log\left({\hat{y}}_{i,c}\right)$$

$y_{i,c}$ is the value of the true label $y_{i}$ in category *c* (one-hot vector, i.e., 1 for the correct category and 0 for the rest). ${\hat{y}}_{i,c}$ is the probability that the sample *i* predicted by the model belongs to category *c*.

For binary classification tasks, the cross-entropy loss function is usually simplified as follows:

(10) $$L=-\frac{1}{n}\sum_{i=1}^{n}\left[y_{i}\log\left({\hat{y}}_{i}\right)+\left(1-y_{i}\right)\log\left(1-{\hat{y}}_{i}\right)\right]$$

$y_{i}$ is the true label of sample *i* (0 or 1). ${\hat{y}}_{i}$ is the probability that sample *i* belongs to category 1 as predicted by the model. This form is also commonly referred to as log loss or binary cross-entropy loss.

### 4.5. Experimental Setup

#### 4.5.1. Experimental Environment

We used the Python-language-based deep learning framework Pytorch to build and train the model; the specific experimental environment is shown in Table 1.

#### 4.5.2. Model Training

For each data set, we ran multiple training sessions, each using K-fold cross-validation to evaluate and observe the training results of each epoch. We chose K values between 10 and 20 (10≤K≤20). For data sets that were not pre-divided into training and test sets, we divided them in a 7:3 ratio, i.e., 70% of the data were used for training and 30% for testing. The individual epoch time and the inference time for a single sample for each data set for model training are shown in Table 2.

We used Gelu as the activation function. During model training, the dropout discard rate was set to 0.3. In addition, we used a training strategy of randomly discarding sub-sequences, again with a discard rate of 0.3. This means that in each training session, we randomly discarded 30% of the sub-sequences. The purpose of this strategy was to avoid overfitting, while hoping that the model would try to learn and predict labels from only some of the features. In this way, we were able to allow the model to learn more robust features during training, thus improving the generalisation ability and robustness of the model. The AdamW optimiser was selected for tuning the model training, and the learning rate was set to 0.001. With these measures, MTC-Net achieved excellent training results on different data sets, proving the effectiveness of our work.

#### 4.5.3. Experimental Results

We conducted two types of experiments: biclassification and multi-classification. In biclassification experiments, the model determines whether a sample is normal or abnormal, while in multi-classification experiments, the model is further subdivided to determine the specific classes of the sample, including normal classes and different types of attacks. We conducted experiments on four widely used network intrusion traffic data sets, including KDD Cup 99, NSL-KDD, UNSW-NB15, and CIC-IDS2017. For the anomaly detection task, where the goal was to distinguish between normal traffic or attacks, Table 3 shows the accuracy, precision, recall, and F1 values achieved by MTC-Net on these four data sets for the performance results.

**Figure 2:**
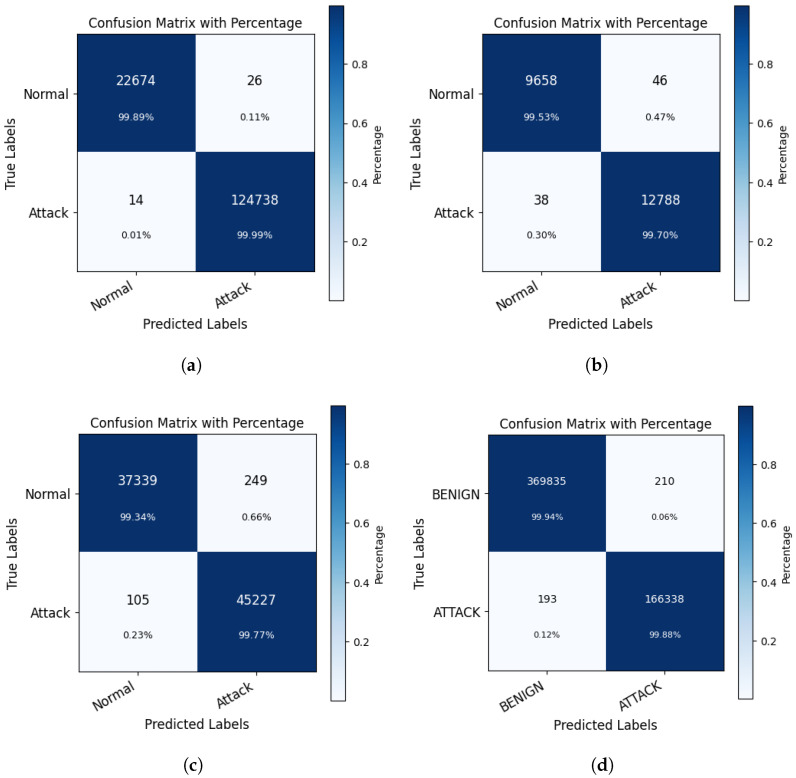
Confusion matrices for anomaly detection (binary classification) results. (**a**) KDD99 data set anomaly detection results. (**b**) NSL-KDD data set anomaly detection results. (**c**) UNSW-NB15 data set anomaly detection results. (**d**) CIC-IDS2017 data set anomaly detection results.

To demonstrate the anomaly detection results of our model more intuitively, we plotted the confusion matrices of the binary classification results of the test sets on the four data sets of KDD Cup 99, NSL-KDD, UNSW-NB15, and CIC-IDS2017, as shown in Figure 2.

**Figure 3:**
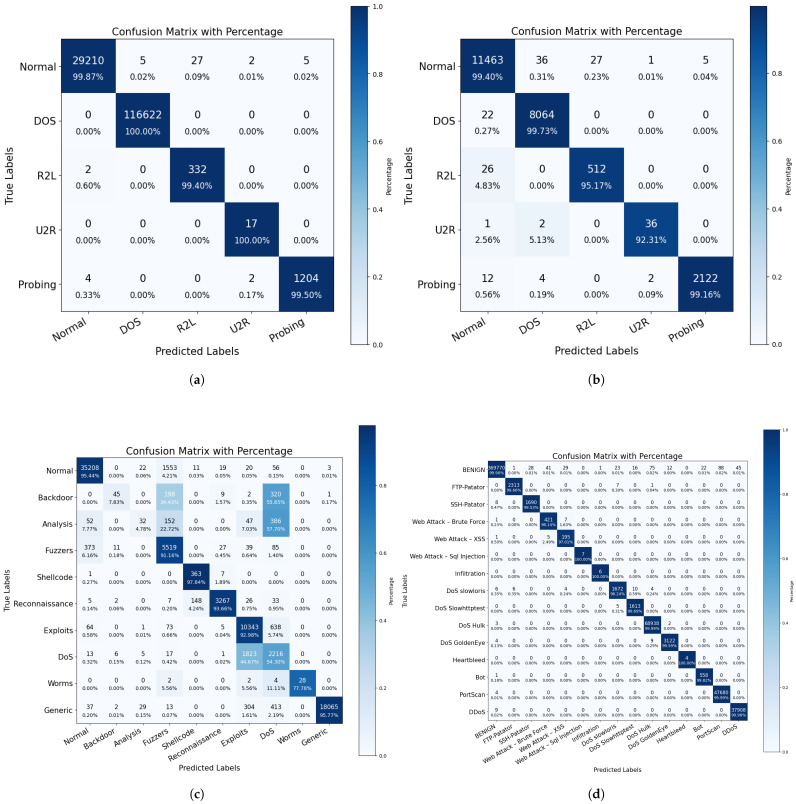
Confusion matrix for multi-classification results. (**a**) KDD99 data set multi-classification results. (**b**) NSL-KDD data set multi-classification results. (**c**) UNSW-NB15 data set multi-classification results. (**d**) CIC-IDS2017 data set multi-classification results.

Furthermore, we conducted multi-classification experiments on these data sets and present the accuracy performance on each data set in Table 4. For the results of the multi-classification experiments on the test set, we also plotted the confusion matrix, as shown in Figure 3. It can be observed that the classification results of MTC-Net are concentrated on the diagonal line, indicating that our multi-classification achieved excellent performance.

Table 5 and Table 6 present the results of our multi-category detection on the KDD Cup 99 and NSL-KDD data sets. The tables include precision, recall, and F1 scores for each specific category.

Table 7 and Table 8 present the multi-classification results obtained on the UNSW-NB15 and CIC-IDS2017 data sets, respectively. The tables include precision, recall, and F1 scores for each specific category.

In order to demonstrate the superiority of MTC-Net, a comparison will be made between MTC-Net and traditional statistical machine learning methods, as well as some deep learning methods proposed in previous research works. Table 9 presents the performance of MTC-Net on the UNSW-NB15 data set in comparison to that of other methods. The experimental results demonstrate that the MTC-Net model achieves the highest performance on the UNSW-NB15 data set, outperforming the other methods mentioned above. Furthermore, the performance of the multi-categorisation task was compared with that of previous works on the KDD99 and NSL-KDD data sets, as shown in Table 10 and Table 11. The results demonstrate that the multi-classification performance of MTC-Net on both data sets yields superior detection results compared to those of the comparison models.

### 4.6. Ablation Study

**Figure 4:**
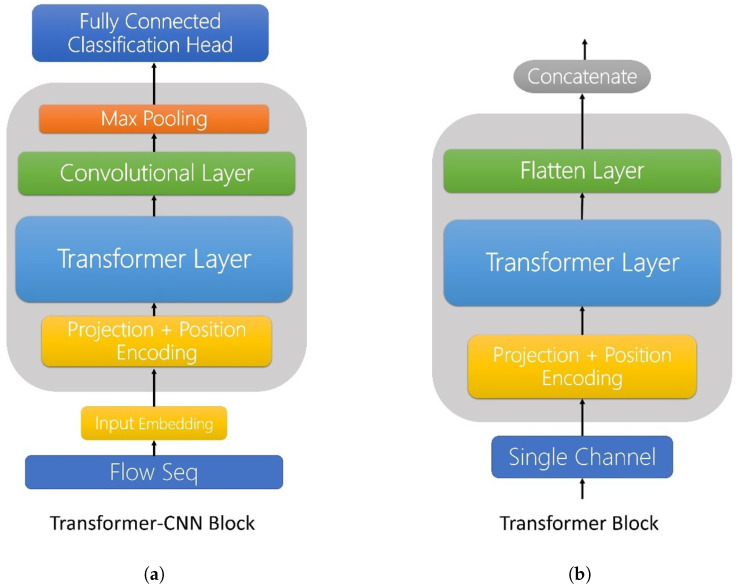
Two comparative models for ablation experiments. (**a**) Direct input to the Transformer without splitting channels. (**b**) Replacement of the CNN of the transfer component block with a Flatten layer.

Ablation experiments were conducted on the UNSW-NB15 data set to verify the effectiveness of the multi-channel strategy. The comparison models are presented in Figure 4, specifically the Transformer–CNN model without splitting channels, as shown in Figure 4a, and the model without using CNN dimensionality reduction to extract features, but instead using a Flatten layer, as shown in Figure 4b.

The experimental results are presented in Table 12. The experimental results demonstrate that when the entire sequence is directly input into the model without splitting the multiple channels, the model is susceptible to biasing the features of the samples with a greater amount of data during the training process. This results in the model exhibiting a tendency to favour certain features in its predictions. In contrast, our multi-channel model, which divides the channels and ensures that each channel is modelled independently, is better able to learn features with robustness, thus avoiding a significant bias in the results. In the model lacking convolutional neural network (CNN) dimensionality reduction for feature extraction, all evaluation metrics are markedly diminished, indicating that the model is unable to effectively capture and compress feature information.

In contrast, the multi-channel model effectively mitigates the effect of data imbalance on the model by separating the channels and utilising convolutional neural networks (CNNs) for dimensionality reduction for feature extraction, thus improving the robustness and generalisation ability of the model. The experimental results demonstrate that the multi-channel model exhibits superior stability and performance across all evaluation metrics, outperforming other comparison models.

In conclusion, the performance of the multi-channel model on the UNSW-NB15 data set verifies its effectiveness and superiority, and further proves the applicability of the design idea of extracting features by separating different feature channels and combining them with CNN dimensionality reduction on complex data sets.

## 5. Conclusions and Future Work

In this paper, we study the problem of network traffic anomaly detection and propose a novel network traffic anomaly detection model, MTC-Net, based on a multi-channel policy and a Transformer–CNN backbone network. Previous studies have focused on individual traffic flows, ignoring the sequential nature of the flows, whereas our approach treats sequential traffic flows within a window as a complete sequential input model. By considering the sequential nature of flows, the model is able to incorporate contextual information to determine traffic classes more accurately. The proposed multi-channel separation strategy enables Transformer to learn finer features in the multi-head self-attention layer, which significantly improves the model’s performance in anomaly detection compared to the state-of-the-art models.

In our future research, we intend to explore the following aspects:

1. Model optimisation and improvement to further optimise the MTC-Net model and explore more feature extraction and fusion methods to improve the accuracy and efficiency of anomaly detection;
2. Real-time detection applications to apply the MTCNet model to real network environments to investigate its performance in real-time traffic detection;
3. Testing on different data sets to test the model on more different complex data sets to verify its generalisation ability in different scenarios;
4. Exploration of hybrid models to combine other deep learning methods with traditional machine learning methods to further improve the comprehensive performance of the model.

## Figures and Tables

**Table 1:** Experimental environment.

Operating System	Ubuntu 20.04.6 LTS
GPU	NVIDIA T4 15GB × 2
Language	Python 3.10
Packages	Pytorch = 2.1.2, Numpy = 1.26.4

**Table 2:** Model runtime.

Data Set	Training Time (An Epoch)	Testing Time (A Sample)
KDD Cup 99	53 s	0.045 ms
NSL-KDD	3 s	0.046 ms
UNSW-NB15	44 s	0.049 ms
CIC-IDS2017	2 min 45 s	0.048 ms

**Table 3:** Anomaly detection performance of MTC-Net on KDD Cup 99, NSL-KDD, UNSW-NB15 and CIC-IDS2017 data sets.

Data Set	Accuracy	Precision	Recall	F1-Score
KDD Cup 99	99.97%	99.95%	99.93%	99.94%
NSL-KDD	99.62%	99.62%	99.61%	99.62%
UNSW-NB15	99.57%	99.58%	99.55%	99.57%
CIC-IDS2017	99.92%	99.91%	99.91%	99.91%

**Table 4:** Accuracy of MTC-Net on KDD Cup 99, NSL-KDD, UNSW-NB15, and CIC-IDS2017 data sets.

	KDD Cup 99	NSL-KDD	UNSW-NB15	CIC-IDS2017
Accuracy	99.96%	99.38%	91.39%	99.91%

**Table 5:** KDD99 Data set multi-class classification results.

Class	Precision	Recall	F1 Score
Normal	99.98%	99.86%	99.92%
DOS	99.99%	100%	99.99%
R2L	92.47%	99.40%	95.81%
U2R	80.95%	100%	89.47%
Probing	99.58%	99.50%	99.54%

**Table 6:** NSLKDD data set multi-class classification results.

Class	Precision	Recall	F1 Score
Normal	99.47%	99.40%	99.43%
DOS	99.48%	99.73%	99.60%
R2L	94.99%	95.16%	95.07%
U2R	92.30%	92.30%	92.30%
Probing	99.76%	99.15%	99.46%

**Table 7:** UNSW-NB15 data set multi-class classification results.

Class	Precision	Recall	F1 Score
Normal	98.47%	95.43%	96.93%
Backdoor	68.18%	7.83%	14.04%
Analysis	35.95%	4.78%	8.44%
Fuzzers	73.25%	91.16%	81.23%
Shellcode	69.54%	97.84%	81.29%
Reconnaissance	97.96%	90.91%	94.29%
Exploits	82.05%	92.97%	87.17%
DoS	53.38%	54.30%	53.83%
Worms	100.00%	77.80%	87.50%
Generic	99.98%	95.76%	97.82%

**Table 8:** CIC-IDS2017 data set multi-class classification results.

Class	Precision	Recall	F1 Score
BENIGN	99.99%	99.90%	99.94%
FTP-Patator	99.70%	99.66%	99.68%
SSH-Patator	98.37%	99.53%	98.95%
Web Attack-Brute Force	90.15%	98.14%	93.97%
Web Attack-XSS	82.98%	97.01%	89.45%
Web Attack-Sql Injection	100.00%	100.00%	100.00%
Infiltration	85.71%	100.00%	92.31%
DoS slowloris	97.95%	98.24%	98.09%
DoS Slowhttptest	98.41%	99.69%	99.05%
DoS Hulk	99.87%	99.99%	99.93%
DoS GoldenEye	99.55%	99.59%	99.57%
Heartbleed	100.00%	100.00%	100.00%
Bot	96.21%	99.82%	97.98%
PortScan	99.82%	99.99%	99.90%
DDoS	99.88%	99.98%	99.93%

**Table 9:** Performance comparison of anomaly detection on UNSW-NB15 data set (best results are highlighted in bold).

Methods	Precision	Recall	F1-Score
NB	74.8%	78.5%	73.1%
KNN	83.1%	87.8%	83.6%
DT	87.4%	91.3%	88.6%
RF	87.4%	91.6%	88.6%
SVM	86.0%	90.2%	87.1%
DAGMM [27]	91.3%	90.3%	90.8%
TGAN-AD [28]	94.5%	87.5%	90.5%
MF-Net [29]	91.2%	89.2%	89.5%
CNNLSTM [30]	93.9%	93.9%	93.9%
**MTC-Net**	**99.6%**	**99.6%**	**99.6%**

**Table 10:** Performance comparison of multi-class classification on KDD Cup 99 data set.

Class	Precision			Recall			F1-Score		
	MF-Net	CNNLSTM	MTC-Net	MF-Net	CNNLSTM	MTC-Net	MF-Net	CNNLSTM	MTC-Net
Normal	73.02%	99.86%	99.98%	98.06%	99.67%	99.86%	83.71%	99.77%	99.92%
DOS	70.55%	99.98%	99.99%	99.99%	100%	100%	74.59%	99.98%	99.99%
R2L	38.64%	98.60%	92.47%	7.46%	99.29%	99.40%	12.50%	98.94%	95.81%
U2R	89.16%	50.00%	80.95%	3.81%	50.00%	100%	7.31%	50.00%	89.47%
Probing	99.55%	97.02%	99.58%	97.12%	98.86%	99.50%	98.32%	97.94%	99.54%

**Table 11:** Performance comparison of multi-class classification on NSL-KDD data set.

Class	Precision			Recall			F1-Score		
	MF-Net	CNNLSTM	MTC-Net	MF-Net	CNNLSTM	MTC-Net	MF-Net	CNNLSTM	MTC-Net
Normal	71.43%	99.10%	99.47%	88.91%	99.28%	99.40%	79.22%	99.19%	99.44%
DOS	60.91%	99.65%	99.48%	81.74%	99.43%	99.72%	69.81%	99.54%	99.60%
R2L	61.54%	97.87%	94.99%	19.48%	98.56%	95.16%	20.08%	98.21%	95.07%
U2R	78.37%	66.67%	92.31%	23.42%	23.42%	92.31%	36.06%	63.16%	92.31%
Probing	88.70%	95.30%	99.76%	75.48%	92.59%	99.15%	81.56%	93.93%	99.46%

**Table 12:** Anomaly detection results of ablation experiments on the UNSW-NB15 data set.

Model	Accuracy	Precision	Recall	F1 Score
Transformer–CNN (No Split Channel)	91.98%	93.36%	91.98%	92.66%
Multi Channel Transformer (No CNN)	97.52%	97.56%	97.52%	97.54%
Multi-Channel Transformer–CNN	99.57%	99.58%	99.55%	99.57%

## Data Availability

The data sets used in this study are all publicly available and can be accessed directly from the web. KDD Cup 99 (accessed on 8 October 2024). NSL-KDD (accessed on 6 October 2024). UNSW-NB15 (accessed on 6 October 2024). CIC-IDS2017 (accessed on 6 October 2024).
